# cGRNB: a web server for building combinatorial gene regulatory networks through integrated engineering of seed-matching sequence information and gene expression datasets

**DOI:** 10.1186/1752-0509-7-S2-S7

**Published:** 2013-10-14

**Authors:** Huayong Xu, Hui Yu, Kang Tu, Qianqian Shi, Chaochun Wei, Yuan-Yuan Li, Yi-Xue Li

**Affiliations:** 1 School of Life Sciences and Biotechnology, Shanghai Jiao Tong University,100 Dongchuan Road, Shanhgai 200240, P.R.China; 2 Shanghai Center for Bioinformation Technology, 1278 Keyuan Road, Shanghai 201203, P.R.China; 3 Key Lab of Systems Biology, Shanghai Institutes for Biological Sciences, Chinese Academy of Sciences, 320 Yueyang Road, Shanghai 200031, P.R.China

## Abstract

**Background:**

We are witnessing rapid progress in the development of methodologies for building the combinatorial gene regulatory networks involving both TFs (Transcription Factors) and miRNAs (microRNAs). There are a few tools available to do these jobs but most of them are not easy to use and not accessible online. A web server is especially needed in order to allow users to upload experimental expression datasets and build combinatorial regulatory networks corresponding to their particular contexts.

**Methods:**

In this work, we compiled putative TF-gene, miRNA-gene and TF-miRNA regulatory relationships from forward-engineering pipelines and curated them as built-in data libraries. We streamlined the R codes of our two separate forward-and-reverse engineering algorithms for combinatorial gene regulatory network construction and formalized them as two major functional modules. As a result, we released the cGRNB (combinatorial Gene Regulatory Networks Builder): a web server for constructing combinatorial gene regulatory networks through integrated engineering of seed-matching sequence information and gene expression datasets. The cGRNB enables two major network-building modules, one for MPGE (miRNA-perturbed gene expression) datasets and the other for parallel miRNA/mRNA expression datasets. A miRNA-centered two-layer combinatorial regulatory cascade is the output of the first module and a comprehensive genome-wide network involving all three types of combinatorial regulations (TF-gene, TF-miRNA, and miRNA-gene) are the output of the second module.

**Conclusions:**

In this article we propose cGRNB, a web server for building combinatorial gene regulatory networks through integrated engineering of seed-matching sequence information and gene expression datasets. Since parallel miRNA/mRNA expression datasets are rapidly accumulated by the advance of next-generation sequencing techniques, cGRNB will be very useful tool for researchers to build combinatorial gene regulatory networks based on expression datasets. The cGRNB web-server is free and available online at http://www.scbit.org/cgrnb.

## Background

Transcription factors (TFs) and micro RNAs (miRNAs) are two primary types of expression regulators for metazoan genomes. They regulate their target genes at the transcription level and the post-transcription level respectively. These two types of regulations interact with each other to modulate celluar processes[1,2] and confer robustness against system perturbation in living organism [3].

**Figure 1:**
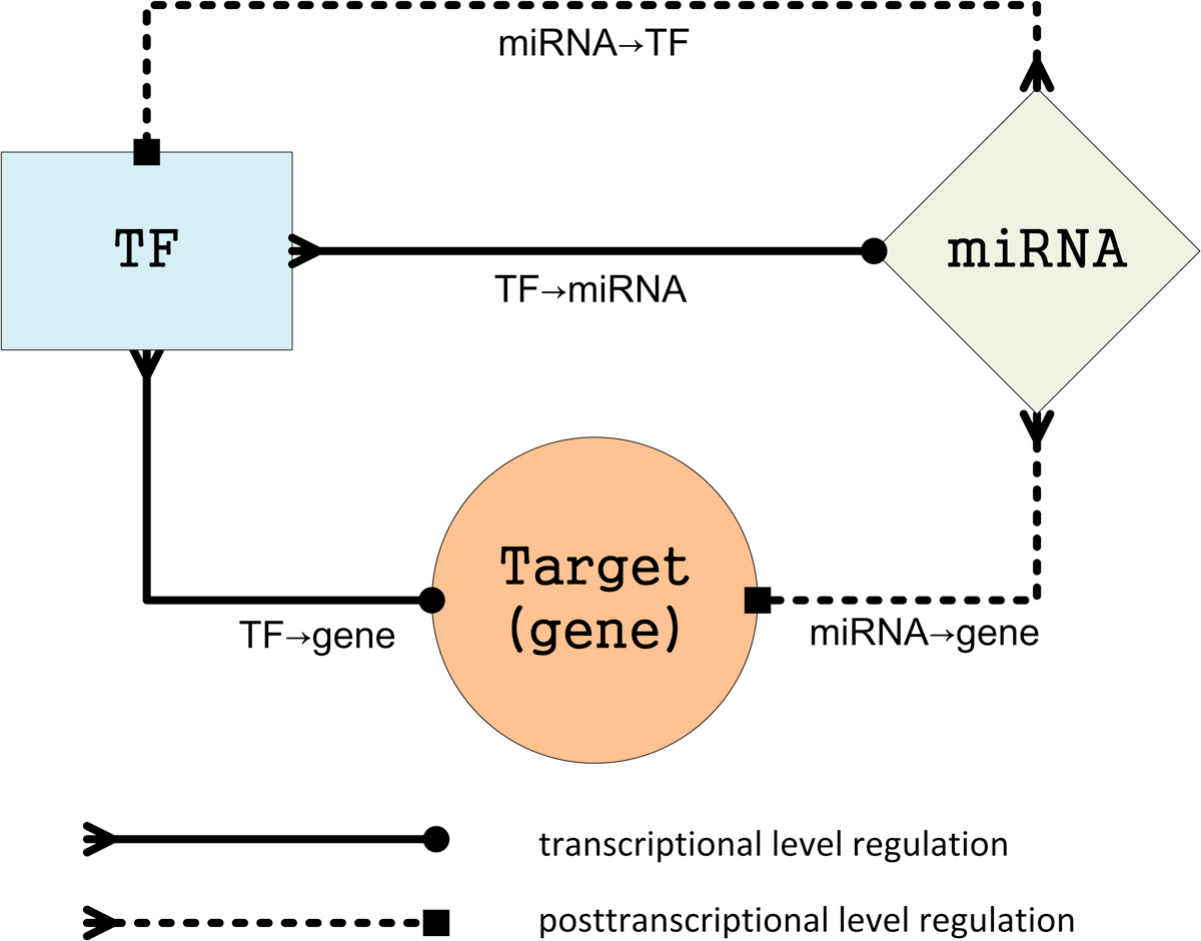
**Basic regulation types in combinatorial gene regulatory network**. TFs and miRNAs are two different types of gene expression regulators that can regulate protein-coding genes (termed "Target (gene)" herein), and they can regulate each other as well. TFs regulate protein-coding genes or miRNA genes at the transcription level (TF→Target (gene) and TF→miRNA, solid lines), while miRNAs regulate protein-coding genes at the post-transcription level (miRNA→Target (gene) and miRNA→TF, dotted lines). In this figure, the same node"Target (gene)" is used to signify both the transcription level and the post-transcription level. As TFs are in fact protein-coding genes, miRNA→target regulations and miRNA→TF regulations can be grouped together into one type miRNA→Target(gene). Overall, the regulations in combinatorial gene regulatory networks are classified into three types: TF→miRNA, miRNA→Target (gene), and TF→Target (gene).

In recent years, great efforts have been devoted to model gene regulatory networks involving TFs and miRNAs (Figure 1). There are many examples in the literatures of 'forward-engineering' works that exploit the complementarity between regulators and their targets to infer putative relationships and then to build combinatorial gene regulation networks [4-6]. In other 'forward and reverse integrated engineering' works [7-9], expression data were integrated with sequence-matching information to build conditional combinatorial gene regulatory networks.

Even though there are a considerable number of methods and algorithms for combinatorial gene regulatory network modelling, most of them have a complex interface and are not easily accessible online. So far, there are only a few resources enabling the forward-engineering curation, such tool as MIR@NT@N (http://maia.uni.lu/mironton.php) [10]. Biologists are increasingly interested in building conditional combinatorial gene regulatory networks based on specific gene expression data, and a web server allowing users to model conditional combinatorial gene regulatory networks from their own gene expression datasets is so valuable. TFactS (http://www.tfacts.org) [8] is a web-based tool of this sort, but it was designed in a simplified fashion with only differentially expressed genes read in; so that, it is difficult to obtain a comprehensive combinatorial gene regulatory networks.

Our group has been working on forward and reverse integrated engineering of combinatorial gene regulatory networks. In our workflow, complementarities information between seed-sequences of regulators and their targets was utilized to generate a reference network which contains all putative regulatory relationships regardless of the spatial/temporal conditions (forward-engineering); expression datasets are used to sift conditional combinatorial regulatory sub-networks from the reference network correlating to particular experiment conditions (reverse-engineering). We have already applied such strategy in two algorithms for building conditional combinatorial gene regulatory networks [11,12].

In order to extend the attainability of these two algorithms and to make them easy to use, we developed cGRNB (combinatorial Gene Regulatory Networks Builder): a web server for constructing combinatorial gene regulatory networks based on user-uploaded expression datasets. We streamlined the R codes of our two separate network builders and formalized them as two functional modules of cGRNB. As the next-generation sequencing platforms are increasingly developing, gene expression datasets, such as the miRNA-perturbed gene expression (MPGE) datasets and the parallel miRNA/mRNA expression dataset, will be generated at an ever-increasing speed. We believe cGRNB is the right tool to enable mining data at this scale and enable advances on conditional combinatorial gene regulatory networks research.

## Methods

### Web server implementation

**Figure 2:**
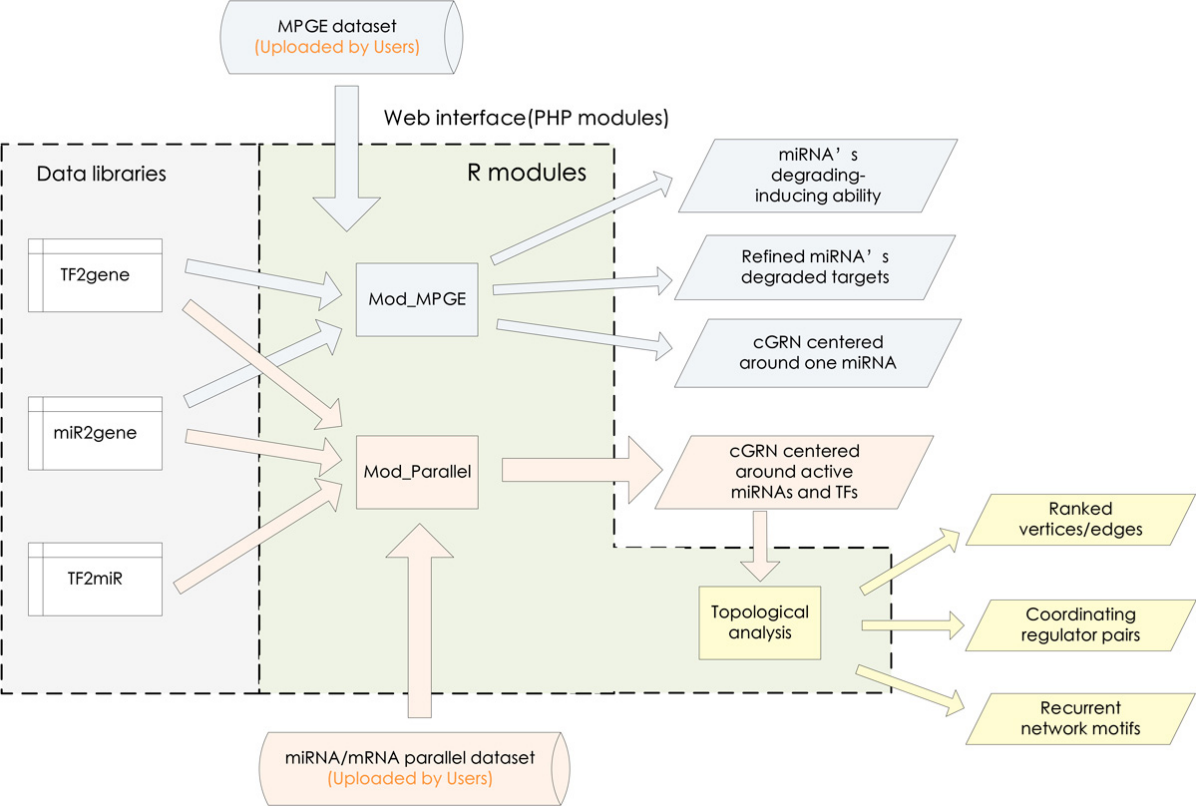
**The framework of cGRNB**. The R statistical environment is employed to carry out two functional modules, Mod_MPGE and Mod_Parallel, based on built-in libraries of regulation relationships (TF2gene, miR2gene and TF2miR) and user-uploaded expression datasets; PHP modules are utilized to deal with data input and output. A web interface is designed in a user-friendly way.

The cGRNB (http://www.scbit.org/cgrnb) is released as a freely accessible tool for modelling combinatorial gene regulatory networks from built-in regulation libraries and users-uploaded gene expression datasets. cGRNB is designed under a PHP and R framework (Figure 2). The PHP modules control the data flow and the R modules perform the calculations.

Two main functional R modules, Mod_MPGE and Mod_Parallel, are deployed in cGRNB. Mod_MPGE works on MPGE datasets to build combinatorial gene regulatory networks covering miRNA-gene and TF-gene regulation. Mod_Parallel works on miRNA/mRNA expression datasets to build combinatorial regulatory networks covering miRNA-gene, TF-gene and TF-miRNA regulations.

**Figure 3:**
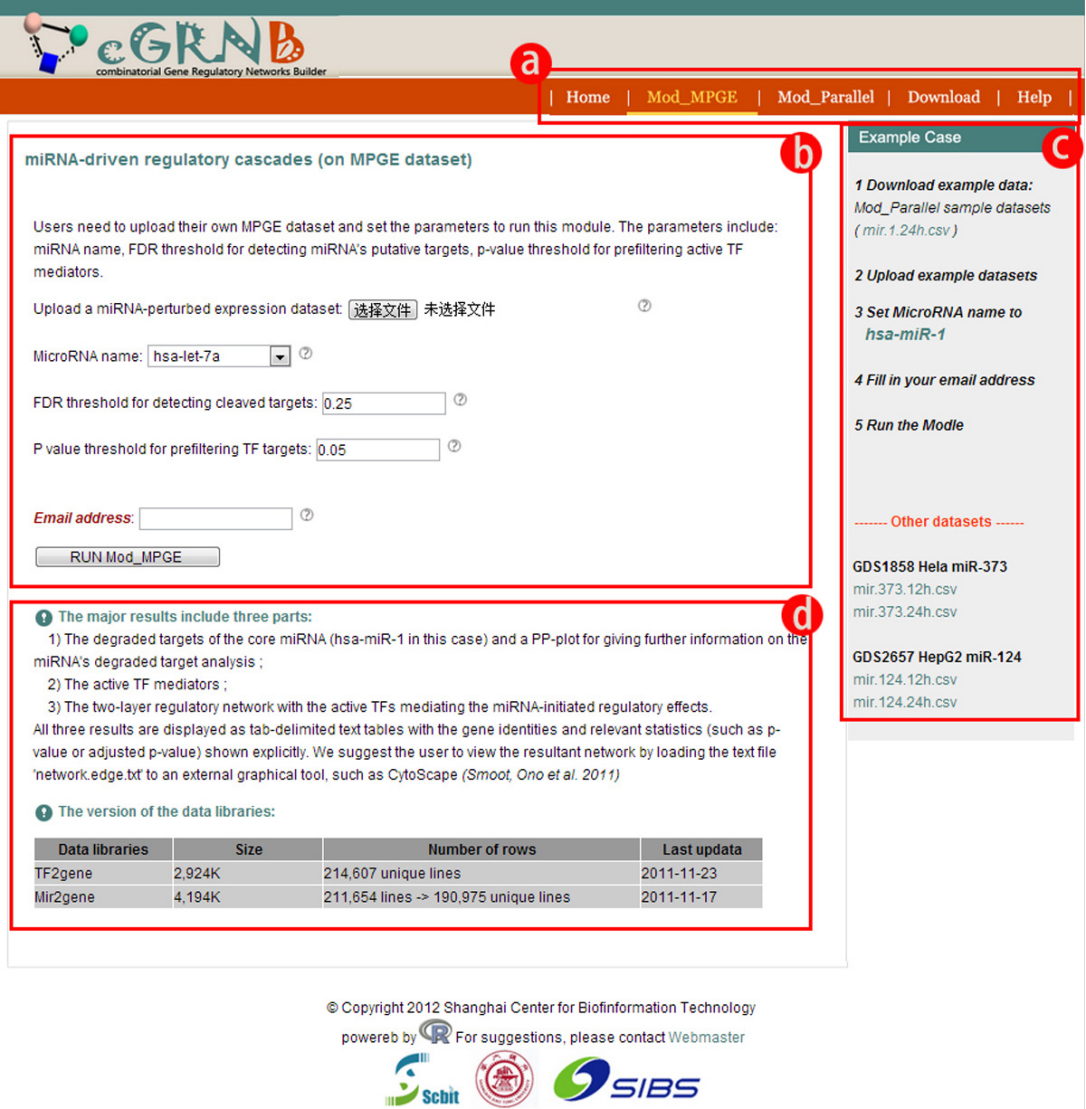
**The screenshots of Mod_MPGE's web page shows how the users interact with the web server**. The web server's interface is neat and user-friendly. a) Navigation bar allows users to jump between functional modules and other facilities; b) At module-invoking area users specify their desired values for parameters of R functional modules and send their execution request; c) Instruction and example block includes a link to a sample dataset with which users can play with the functional modules; d) At information area, users get to know the structure of the module's output and other related information.

The interface is web-based and users without R programming expertise can freely utilize the calculation modules. After the expression datasets are uploaded, users can set the required parameters on the web graphic interface (Figure 3). When the calculation is finished, users can view or download the HTML formatted reports through a URL sent to the pre-designated email addresses.

### Data libraries

There are three data libraries (TF2gene, TF2miR and miR2gene) deposited in cGRNB as built-in components that will be called at every calculation process. The TF2gene and TF2miR libraries are comprised of forward-engineered putative "TF to gene" and "TF to miRNA" regulation relationships respectively. These two libraries were extracted mainly from the source file 'tfbsConsSites.txt' and 'tfbsConsFactors.txt' obtained from UCSC hg19, where the two source files were the results of scanning the human genome for human/mouse/rat conserved TF binding sites (http://genome.ucsc.edu/cgi-bin/hgTables). The miR2gene library, extracted from original dataset of starBase(http://starbase.sysu.edu.cn/) [13], includes putative "miRNA to gene" regulation relationships mapped from CLIP-Seq and Degradome-Seq data. We processed the original data files so that miRNA transcript names are consolidate into their root forms since they are indexed according to their genome coordinates. For example, 'hsa-let-7a-1', 'hsa-let-7a-2' and 'hsa-let-7a-3' are renamed to be 'hsa-let-7a'. TF2miR and miR2gene libraries are also subject to this rule, and we strongly recommend users to process their expression datasets in the same manner. A Perl script tailored to this goal can be found in the download page of cGRNB.

Detailed information about how to process and access these data libraries can also be found at the help page of the web server.

### Mod_MPGE

In an MPGE experiment, a miRNA is first transfected into a certain cell line. After a time period (usually 12h or 24h), the mRNA levels in the miRNA-transfected and pre-transfected cells are both measured and compared. A MPGE dataset can be utilized for building a miRNA-driven two-layer combinatorial gene regulatory network.

**Figure 4:**
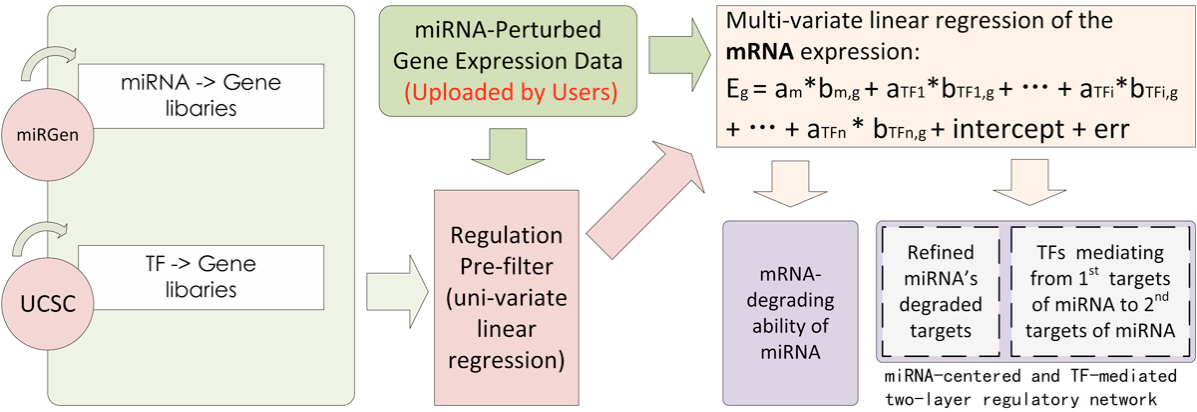
**Overview of the workflow inside Mod_MPGE**. Relying on three inputs (miR2gene, TF2gene, and an MPGE dataset), Mod_MPGE performs a one-sided Kolmogorov-Smirnov (K-S) test to evaluate the significance of miRNA degradating ability in human cells, a non-parametric statistical test to refine miRNA's degraded targets from forward-predicted putative targets, a pre-filtering uni-variate linear regression model to cut down the regulator numbers and an ultimate multi-variate stepwise linear regression model to determine miRNA's downstream TF mediators. Taking the sifted miRNA→gene and TF→gene regulations together, a hypothetical two-layer regulatory network centered on the perturbed miRNA and its downstream mediating TFs is mapped. In the formulae, *E_g _*stands for the mRNA level of a target gene *g*; *b_m,g _*and *b_TFi,g _*, with values zero or one, encode the putative regulatory relationships between *g *and miRNA *m*, and *g *and *TF*_*i*_, respectively; *a_m _*and *a_TFi _*quantifies the purturbed miRNA's and a TF's regulating coefficients (regulatory strength), which are to be determined through the linear regression modelling.

Based on the MPGE dataset and two data libraries (miR2gene and TF2gene), Mod_MPGE is aimed at three mutually related goals and in the end arrives at a two-layer regulatory networks centring on the perturbing miRNA and its downstream regulating TFs (Figure 4). The three goals are as follows: 1) to evaluate the significance of miRNA degradating mRNAs in human cells; 2) to refine miRNA's degraded targets from forward-predicted putative targets; 3) to identify mediating TFs that transfer miRNA's regulation effect to downstream secondary targets. Goal one and goal two are achieved through non-parametric statistical tests that compare the mRNA level(s) of miRNA's putative targets against those of the non-targets (the complement set to the putative targets). For goal three, we first perform a pre-filtering uni-variate linear regression to screen out highly plausible regulator-target relations one by one, and then apply a multi-variate linear regression to further refine the combinatorial regulators of each target. Been taken together, the miRNA2gene links and TF2gene links output from goal two and goal three make up a two-layer regulatory network centring on the perturbed miRNA and its downstream mediating TFs. The overall diagram of the Mod_MPGE algorithm is illustrated in Figure 4 and the full mathematics details can be found in our previous related algorithm paper [11].

### A case study of Mod_MPGE

We tested Mod_MPGE with an MPGE dataset related with "hsa-miR-1" miRNA. This MPGE dataset GDS1858 was obtained from Gene Expression Omnibus (http://www.ncbi.nlm.nih.gov/sites/GDSbrowser?acc=GDS1858). It contains the expression log ratios of a total of 20,127 protein-coding genes, which are obtained by comparing the expression profiles of HeLa cells before and after the transfection of hsa-miR-1. We recommend the tabular data of the MPGE dataset to be stored in a CSV (Comma-Separated Values) file. Spreadsheet software like Microsoft Excel will enable CSV extension conversion. In the CSV file, the first line contains a description of the columns; all other lines must contain a gene symbol and an expression log ratio in the first and second column separated with a comma. The only procedure to begin a calculation is to upload the MPGE data file to the server, set the appropriate parameters, provide a valid e-mail address and click the run button. The job runs in the web server and an e-mail including the URL of the report page is immediately sent to the user. In this example, it took about 5 minutes to finish the calculation and get the result report.

The result include three sections: 1) the targets of the particular miRNA (hsa-miR-1 in this case) and a PP-plot chart with detailed information on the miRNA's target analysis; 2) a list of the TF mediators; 3) a two-layer combinatorial gene regulatory network with the TFs mediating and the miRNA-initiating regulatory effects. Most of the results are shown as tab-delimited text tables with the gene identities and relevant statistics (http://www.scbit.org/cgrnb/doR_target_result.php?jobID=821338391035). As R is not good at displaying a dynamic graphic object on the web interface, we suggest users to download the original CVS file 'network.edge.txt' from the report page and reload it to an external graphical tool, for example CytoScape [14].

### Mod_Parallel

A parallel miRNA and mRNA expression dataset includes two data matrices of the same set of column headers (experimental conditions) but different sets of row headers (biological molecules) - one set for miRNAs and the other for mRNAs. A parallel miRNA and mRNA expression dataset can be utilized for building a combinatorial gene regulatory network encompassing three types of gene regulations ("TF to gene", "TF to miRNA" and "miRNA to gene").

**Figure 5:**
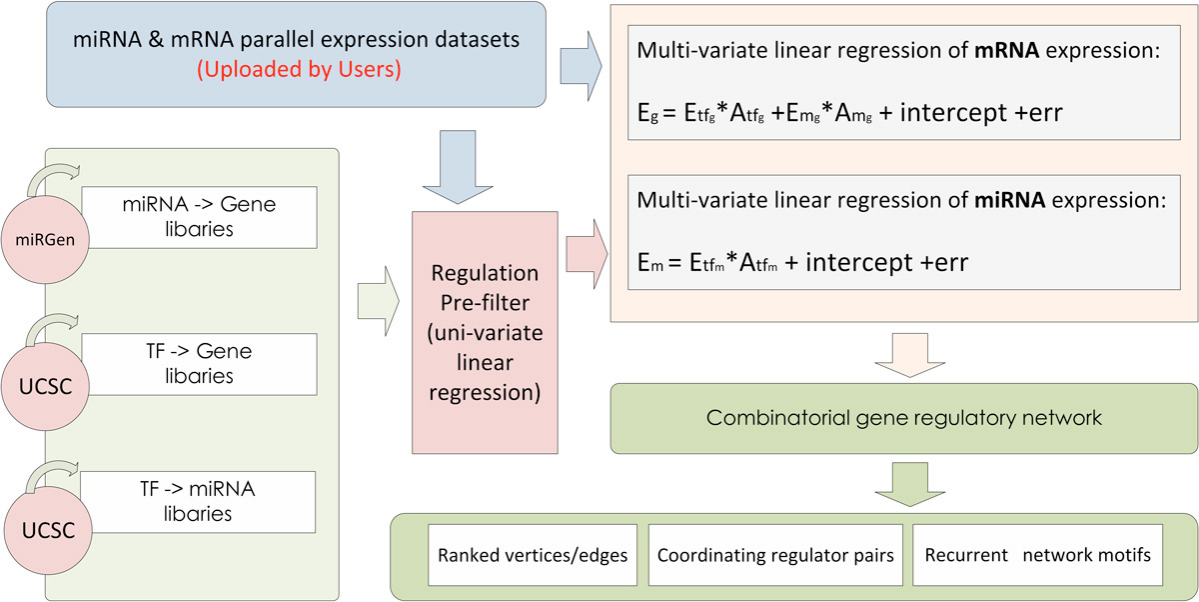
**Overview of the workflow inside Mod_Parallel**. Relying on four inputs (miR2gene, TF2gene, TF2miRNA, and a miR/mRNA parallel expression dataset), Mod_Parallel decides significant miRNA-gene and TF-gene regulations from mRNA expression dataset and significant TF-miRNA regulations from miRNA expression dataset through two multi-variate linear regression models. The reserved regulations of three different types are combined to form a comprehensive TF-and-miRNA-involving combinatorial gene regulatory network. Topological analyses of the combinatorial network are further carried out which yield single regulators, regulator pairs, and triangle regulation motifs of remakable notice. In the formula, *E_g _*and *E_m _*stand for the mRNA level of a target protein-coding gene *g *and a miRNA *m *respectively; *E_tfg_*, *E_mg_*, and *E_tfm _*are vector-form notations of the transcript profiles of *g*'s regulating TFs, *g*'s regulating miRNAs, and *m*'s regulating TFs; *E_tfg_*, *E_mg_*, and *E_tfm _*quantify the to-be-estimated regulating coefficients (in vector forms) of the same three sets of regulators.

Based on the parallel miRNA/mRNA expression dataset and three data libraries (TF2gene, miR2gene and TF2miR), Mod_Parallel sets out to map a comprehensive TF-and-miRNA-involving combinatorial gene regulatory network and this also goes further to analyze its various topological properties (Figure 5). Similar to Mod_MPGE, here the multi-variate linear regression model is adopted to infer plausible regulation relationships. In this module, because heterogeneous expression data types (miRNA expression and mRNA expression) are available, we build up two multi-variate linear equations to model the expression of mRNAs and miRNAs separately. TF-gene and miRNA-gene regulations are output of the mRNA-targeted equation, and TF-miRNA regulations are output of the miRNA-targeted equation. Been taken together, the three types of regulations made up a comprehensive combinatorial gene regulatory network correlating to the particular experimental conditions.

Mod_Parallel then conducts topological investigation of the resultant combinatorial regulatory network and identifies the important vertices/edges, regulator pairs and three-vertex regulating motifs. We first pinpoint the crucial vertices and edges of the network according to degree rank and betweenness rank. Then the 'co-regulating regulator pairs' in the network is marked. To this end, we check all regulator pairs by testing the significance of their potential co-regulating targets. Finally we carry out the triple-vertex motif analysis. Theoretically, there are eighteen triple-vertex regulatory motifs involving at least one miRNA and one TF. These motifs are defined as closed triple-vertex regulatory circuits, and can be classified into 'feed-forward loops' (FFLs) and 'feed-backward-loops' (FBLs) according to the ways of the directional regulations being connected. We count the occurrences of all possible triple-vertex motifs in the resulting network and estimate the corresponding p-values by comparing the real occurrence against the counterpart occurrences in the randomly shuffled networks.

While the overall diagram of the Mod_Parallel algorithm is illustrated in Figure 5, more details of the algorithm can be found in our previous algorithm paper [12].

### A case study of Mod_Parallel

The parallel cancer gene expression datasets were downloaded from CellMiner (http://discover.nci.nih.gov/cellminer/loadDownload.do). The two datasets, one for miRNAs and the other for mRNAs, were designed to study a total of 60 types of human cancer cell lines. The experiments were carried out on the 41,000-probe Agilent Whole Human Genome Oligo Microarray and the 15,000-feature Agilent Human microRNA Microarray V2.

The miRNA expression dataset included 365 human miRNAs with detectable expression levels [13]. After miRNA names and their corresponding data were pre-processed, this dataset covered only 266 miRNAs. The mRNA expression dataset had more than 41,000 data rows, while 40,155 rows have Entrez GeneID available. After removing data rows without GeneID and combining rows for identical genes, we finally obtained the expression data for 21,319 protein-coding genes. These two parallel expression datasets are taken as sample datasets for Mod_Parallel, which can be obtained from the cGRNB's "Download" page. The calculation takes around 60 minutes on our web server.

The results of Mod_Parallel include four parts: 1) a CSV file that indicates the edges of the combinatorial gene regulatory network; 2) the vertices/edges ranked by topological features; 3) significantly co-regulating regulator pairs (*p *< 0.01, one-sided Fisher's exact test); 4) significance of the recurrence of the 18 triple-vertex motifs and all instances of the existing motifs. The results are provided as tab-delimited tables and can be downloaded as CSV formatted files from the example report (http://www.scbit.org/cgrnb/doR_network_result.php?jobID=931338180093).

## Discussion and conclusion

Nowadays a promising technique in computational biology is engineering conditional combinatorial gene regulatory network that represents specific biological contexts through integrating heterogeneous data, such as sequence data and expression data. In this paper, we report cGRNB, a web server for modelling combinatorial gene regulatory networks through integrated engineering of seed-matching sequence information and gene expression datasets. The cGRNB fills in the blank of easy-to-use combinatorial gene regulatory network generator in public domain.

Currently, the cGRNB has two functional components that deal with MPGE datasets and parallel miRNA/mRNA expression datasets separately. We will update the built-in data libraries regularly to keep up with the respective source files. Since the next-generation sequencing techniques are increasingly accessible, more and more parallel miRNA and mRNA expression datasets will be available. It is our belief that our web server will greatly help biologists to model and analyze conditional combinatorial gene regulatory networks based on their own expression datasets.

## Competing interests

The authors declare that they have no competing interests.

## Authors' contributions

HX and HY designed the framework of the whole web server and devised the initial model. HX created the webserver and implemented PHP modules working together with the R modules. HY and KT developed the original R modules. HX and HY drafted and revised the manuscript. QS performed the case studies with the sample datasets. CW contributed discussions, and revised the manuscript. YXL and YYL devised the study, drafted and revised the manuscript. All authors have read and approved the final manuscript.

## Declarations

The publication of this article was funded by the National key basic research program (funding numbers: 2011CB910204, 2012CB316501, 2011CB910200, 2010CB912702); Chinese Academy of Sciences (Innovation funding KSCX2-YW-R-112, KSCX2-EW-R-04); National Natural Science Foundation of China (31000380 to HY, 60970050 to CW, 31171268 to LYY, 90913009 to YXL).

This article has been published as part of *BMC Systems Biology *Volume 7 Supplement 2, 2013: Selected articles from The 6th International Conference of Computational Biology. The full contents of the supplement are available online at http://www.biomedcentral.com/bmcsystbiol/supplements/7/S2.
